# Advances in diamond nanofabrication for ultrasensitive devices

**DOI:** 10.1038/micronano.2017.61

**Published:** 2017-10-23

**Authors:** Stefania Castelletto, Lorenzo Rosa, Jonathan Blackledge, Mohammed Zaher Al Abri, Albert Boretti

**Affiliations:** 1 School of Engineering, RMIT University, Bundoora, Victoria 3083, Australia; 2 Swinburne University of Technology, Centre for Micro-Photonics (H74), Hawthorn, Victoria 3122, Australia; 3 Department of Information Engineering, University of Parma, Parma 43121, Italy; 4 Military Technological College, Muscat 111, Sultanate of Oman; 5 Dublin Institute of Technology, Rathmines Road, Dublin 6, Ireland; 6 Department of Petroleum and Chemical Engineering, Sultan Qaboos University, PO Box 33, Al-Khoud, Muscat 123, Sultanate of Oman; 7 Water Research Center, Sultan Qaboos University, PO Box 17, Al-Khoud, Muscat 123, Sultanate of Oman; 8 Department of Mechanical and Aerospace Engineering, Benjamin M. Statler College of Engineering and Mineral Resources, West Virginia University, P.O. Box 6106, 325 Engineering Sciences Building, Morgantown, WV 26506, USA

**Keywords:** nano-diamonds, nanofabrication, nanomechanics, nanophotonics, optomechanics

## Abstract

This paper reviews some of the major recent advances in single-crystal diamond nanofabrication and its impact in nano- and micro-mechanical, nanophotonics and optomechanical components. These constituents of integrated devices incorporating specific dopants in the material provide the capacity to enhance the sensitivity in detecting mass and forces as well as magnetic field down to quantum mechanical limits and will lead pioneering innovations in ultrasensitive sensing and precision measurements in the realm of the medical sciences, quantum sciences and related technologies.

## Introduction

Diamond is an ideal platform for nano- and microfabrication leading to a range of robust sensors. This is due to the outstanding mechanical and wide spectral optical transparency of diamond, combined with biocompatibility and lack of toxicity in both bulk and nanostructures. Moreover, diamond can be fabricated with high purity and control using chemical vapor deposition. However, due to its hardness and cost, some of its applications in nanomechanics, optomechanics and nanophotonics reside mainly in its polycrystalline or nanocrystalline forms, which do not always retain the nuance of the outstanding properties of monocrystalline diamond.

A recent review^1^ describes diamond optical and mechanical properties compared to other materials currently used for optomechanical components (Si, Si_3_N_4_, SiC, and α-Al_2_O_3_), showing that, in principle, there are more favorable properties of diamond for nanomechanical and optomechanical realizations. Therefore, a considerable effort has been taking place over the last five years to exploit these properties in a wide spectrum of diamond nanosystems. To date, single crystal diamond utilization for nanomechanical components^2^, (for example, nano electro-mechanical systems (NEMS) and micro electro-mechanical systems (MEMS)), as well for nanophotonics^3,4^ compared to above mentioned materials, has been limited due to its growth requirements. In fact, a single crystal diamond cannot be grown on any other substrate than itself. This prevents wafer-scale processing and it represents a challenge in the application of conventional diamond nanofabrication methods. On the other hand, polycrystalline diamond films, which can be grown in high quality from 4 to 6 inches’ wafers^5^, permit to fabricate opto-mechanical components with quality factors and oscillation frequencies rivaling single crystal diamond^6^.

Thus, diamond nanomechanical systems, such as nanomechanical resonators and nanocantilevers, can provide the means for ultrasensitivity in detecting nanomechanical motions in force microscopy^7^, and to establish optomechanical systems^6^, where optical modes and mechanical modes can be coupled. Furthermore, nanofabrication has led to photonic components in diamond, such as waveguides, photonic crystals (PHC) and optical nanoresonators, to advance light manipulation, propagation and confinement^8–10^, while their combination with mechanical modes can advance cavity optomechanics^11,12^.

Another important feature in diamond is the harboring of isolated atomic color centers that can be used as fluorescent and spin probes for single molecule detection and imaging as well as for fundamental investigation into quantum science. For example, the nitrogen vacancy (NV) center^13,14^ in diamond possesses the exceptional optical signatures of an associated electron spin, observed and subsequently exploited for applications including quantum information processing^15^ and magnetometry^16^, with a prominent application being nano–magnetic resonance imaging (nano-MRI) and microscopy^17^. It has now been established that to reach exceptional sensitivity in diamond devices with integrated fluorescence and spin probes, the main thrust resides in its single crystal nanofabrication and material purity. Many challenges regarding this accomplishment have recently been addressed, and a considerable number of advances in this field have now brought forward representative nano- and micro-systems that promise a paradigm shift in sensing, and to a certain extent surpasses other conventional micro- and nano-devices developed years ago.

Proof of the principal demonstrations of integration of these diamond probes within nanoresonators, based on other outstanding materials more amenable to nanofabrication such as Si_3_N_4_, Si nanophotonics or SiC cantilevers, have shown the possibility to couple nanomechanical and nanophotonic systems with atomic scales probes, such NV center in nano-diamond, bulk diamond or thin diamond membranes (for a review on this specific hybrid systems, see Ref. 18). However, novel nanosystems sculpted directly in diamond with integrated spin and fluorescent atomic probes are an advanced platform for the realization of ultrahigh sensitivity or strong coupling of mechanical, optical and spin modes to coherently transfer information from the atomic level to the macroscopic level in a photonic quantum network made of these components^19^ or for force microscopy and nano-MRI.

In this paper, we focus on a review of the main concepts that lie behind nano-mechanical, nanophotonics and opto-mechanical all-diamond components and recent advances in their fabrication in single crystal diamond, required in the field of ultrahigh-sensitivity sensors. Furthermore, we highlight the nanofabrication of single crystal diamond with integrated spin and optical centers acting as multifunctional atomic size probes for ultrahigh-sensitivity devices and leading to exciting applications in field of quantum science and technology.

## Nanomechanical components

Nanomechanical resonator sensing devices have a very small mass and thus high responsivity to changes in the local environment. Nanomechanical systems are therefore relevant in force measurements via scanning probe magnetic and atomic force microscopy (AFM), yielding present sensitivities at the attonewton (aN) level (~10–18 N Hz^−1/2^) and mass detection of single molecules and proteins^20–23^.

The minimum force detectable by a nanomechanical cantilever, equivalent to a force noise associated with mechanical dissipation, can be calculated if the cantilever behaves as a simple harmonic oscillator^24^. It is limited by thermomechanical noise, which is governed by dissipation of the mechanical energy and sample temperature, and is given by:

$$F_{\min}=\sqrt{\frac{2k_{B}TBk_{c}}{\pi f_{c}\cdot Q_{m}}}$$

Here *k*_c_ is the spring constant, *f*_c_ is the resonator frequency, *B* is the resonator bandwidth and  *Q*_m_ is the mechanical quality factor.  *Q*_m_ characterizes a resonator's bandwidth relative to its center frequency *f*_c_. Higher  *Q*_m_ indicates a lowest rate at which the resonator loses mechanical energy. For a simple rectangular cantilever, this minimum detectable force can also be expressed in terms of the cantilever width (*w*), thickness (*t*), length (*l*), mass density (*ρ*) and Young Modulus *E*, in the case of non-internal stress, as

$$F_{\min}=\sqrt{\frac{wt^{2}k_{B}TB}{lQ_{m}}\sqrt{E\rho }}$$

Therefore, to increase sensitivity, a mass reduction is a suitable solution, in addition to making the cantilever narrow, thin and long. However,  *Q*_m_ needs to be preserved in the scaling of the cantilever, and to this end, many (yet not fully known) factors at the micro- and nanoscale tend to introduce other mechanisms responsible for energy dissipation. Recent progress has consisted in fabricating thinner resonators with lower spring constants *k*_c_ in conventional materials, such as Si^25^, or by changing the resonators geometry using doubly clamped beams^26^. However, reducing the nano-resonators, thickness introduces a decreased *Q*_m_ due to increased surface friction by surface defects^24,25^. *Q*_m_ can be restored by reducing the temperature^27^ (the surface friction being less at cryogenic temperatures), which reduces practicability. Another option is to use materials that naturally lend themselves to high *Q*_m_ and high *f*_c_ (noting that $Q_{m},f_{c}\propto \sqrt{k_{c}};k_{c}\propto E$), due to higher *k*_c_, because of high stiffness (i.e., a high Young Modulus *E*, *E*=1220 GPa and *ρ*=3530 kg m^−3^ for example single crystal diamond)^24^. The same materials are, however, more challenging in establishing micro- and nanofabrication procedures, which can introduce unexpected mechanical losses.

The motivation behind the efforts for fabricating nanomechanical components in diamond is twofold. One is based on its excellent mechanical and thermal properties and the other is related to the sub-bandgap optical and spin-carrying color centers due to a large bandgap (5.47 eV). These color centers facilitate the optical readout of the nano-resonator sensors on one side (the electrical readout in nano-mechanical resonators is more cumbersome than in larger micro mechanical resonators), and they introduce a direct magnetic sensing degree of freedom and can permit optomechanics in the same device. The first motivation relies on the fact that diamond has the largest Young modulus (inferior only to graphene), which is responsible for a high mechanical resonance frequency and low mechanical losses. Furthermore, the high thermal conductivity (2200 W mK^−1^) and low thermal expansion coefficient permit high thermal dissipations. Diamond therefore offers a strategy for exploiting the intrinsically high *f*_c_ and achievable high *Q*_m_=6·10^6^ at the millikelvin level^27^. These properties remain good at room temperature (*Q*_m_=1.2×10^6^), making possible ultrahigh sensitivities without the need of cooling. Combined with diamond biocompatibility, this can extend the above-mentioned applications at the current sensitivity to living cells and tissues by mere substitution of the material for nano-resonators.

The detection of nuclear magnetic resonance signals using spin magnetic resonance force microscopy^28^ requires a sensitivity in the zeptonewton (zN) range (~10^−21^ N Hz^−1/2^) about force detection, which is currently achieved using trapped ions^29^ and nano-tubes mechanical resonators^30^. The integration of spin sensitive color centers in diamond within high-sensitivity single crystal nanomechanical resonators could allow a more practical approach to achieve single nuclear spin sensing at room temperature; this has already been demonstrated in a dilute ensemble of nuclear spins without resorting to the use of ultrasensitive nanomechanical resonators (for a recent review, see Ref. 17 and citations therein).

A competitive material to diamond that is already replacing Si NEMS/MEMS for harsh environments is SiC. NEMS are based on epilayers SiC and have been fabricated for some years but with a lower *Q*_m_=500 than current diamond can realize, due to surface roughness, with a record frequency of *f*_c_=1 GHz (Ref.^31^). It is important to note that a figure of merit which should be considered to enhance sensitivity in nanomechanical resonators is based on the *f*_c_ *· Q*_m_ product. In this respect, in single crystal diamond nanomechanical resonators, the present record achieved is of *f*_c_ *· Q*_m_*=*2.2 THz at 5 K in doubly clamped nano-beam waveguides^32^, while for diamond nanocantilevers *f*_c_ *· Q*_m_*=*48.3 GHz at 3 K (Ref. 27). A more remarkable record is achieved in optomechanical single crystal diamond micro disk resonators operating in ambient condition^12,33^, with *f*_c_ *· Q*_m_*=*19 THz, while similarly *f*_c_ *· Q*_m_*=*9.5 THz at room temperature in polycrystalline 3C-SiC optomechanical micro-resonators has been achieved (the theoretical maximum achievable in SiC being *f*_c_ *· Q*_m_*=*300 THz at room temperature)^34^. It is expected that a similar race could start regarding MEMS/NEMS^35^ in reconsidering conventional materials, hosting color centers as diamond, such as SiC, for applications envisaged for current diamond nanomechanical resonators.

**Figure 1:**
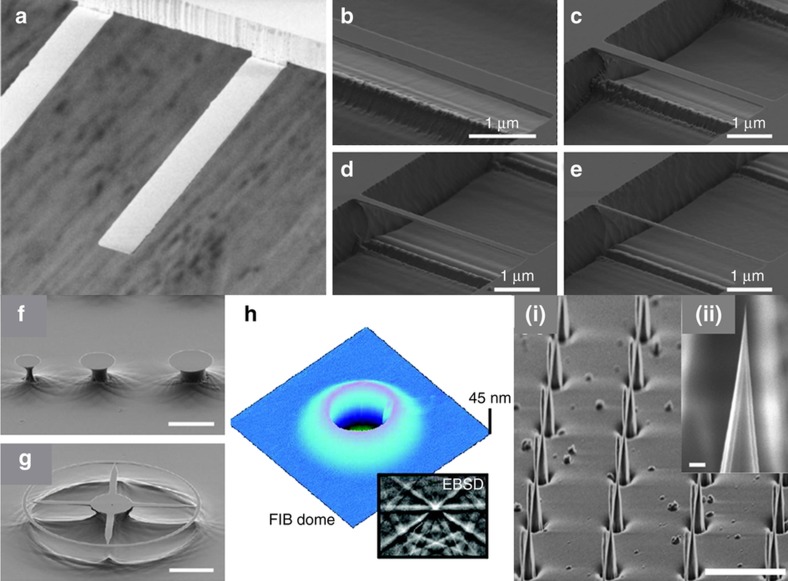
(**a**) Scanning-electron-microscope (SEM) micrographs of 100(25) nm thin and 12 μm wide optical-grade single crystal diamond cantilevers. Image reprinted by permission from Macmillan Publishers Ltd: [Nature Communications]: Ref. 27, copyright 2014. SEM of diamond doubly clumped nanobeams with width (**b**) 500 nm, (**c**) 350 nm, (**d**) 200 nm and (**e**) 75 nm. (**f**) ~3–5 μm diameter undercut micro-disks; and (**g**) ~500 nm wide nano ring structure. Images reprinted (adapted) with permission from: Ref. 26. Copyright 2012 American Chemical Society. (**h**) Atomic force microscopy (AFM) image of a focused-ion-beam (FIB)-defined dome, scan size 5·5 μm^2^. Inset shows an electron backscatter diffraction (EBSD) pattern confirming the single-crystal nature of the device layer. Image reprinted with permission from: Ref. 36. Copyright 2011 American Chemical Society). (i) Regular array of lithographically defined single crystal diamond tips (scale bar, 10 μm) and (ii) zoom in after cleavage resulting in sharp tips with radii of about 10 nm. The scale bar is 100 nm. Images reprinted (adapted) with permission from: Ref. 37. Copyright 2015 American Chemical Society.

Some exemplary common nanomechanical resonators realized in diamond for ultrahigh force sensitivity are shown in Figure 1. They are based on nanocantilevers, which are clamped on one end only (Figure 1a) or nanoscale beams that are clamped on both ends (Figures 1b–e). Centrosymmetric mechanical resonators, such as nano-ring and disk resonators (Figures 1f and g), which are free standing, can be designed to host mechanical and optical resonance at the same time. They have potentially higher optical performance compared to nano-cantilevers once transferred in free-standing integrated optical components. Finally, another nano-mechanical system in single crystal diamond named the ‘dome’ resonator (Figure 1h) appears as a shell-type resonator which is comprised sub-100 nm thickness diamond, and could be used in the development of future nano-electromechanical devices in diamond using grapheme-based electrical components^36^.

In the realm of magnetic force microscopy, the fabrication of single diamond nano-tips or nano-wires^37^ has recently been sought after and has been considered a challenge in the field. Diamond nanotips made of single crystals (i, ii in Figure 1) permit the ability to reach <100 nm thickness in nano-mechanical resonators to enhance scanning probe microscopes in terms of their imaging resolution. Ultimately, the integration of NV-centers in diamond nanotips will provide the ability to achieve higher sensitivity in MRI of an ensemble of nuclear spins even at room temperature.

### Diamond nanocantilevers

The key paradigm shift on the material fabrication relied on novel approaches for cantilever fabrication together with the very recent commercial availability of templates based on optical-grade (doping concentration of Boron and Nitrogen substitutional B, N_s_<5 ppm) and electronic-grade (doping concentration and B, N_s_<5 ppb) single crystal diamond plates of size 5·5·(0.02–0.04) mm^3^, with an initial surface roughness of <5 nm. These commercially available templates have considerable thickness variation of 10 μm over the plate. This requires protocols for template-assisted precision re-polishing to achieve thickness uniformity better than 1 μm, together with methods for handling such thin plates^27,38^. After plate re-polishing, two methods can be used for handling and subsequent etching of the nanocantilevers. One method is based on developing a diamond-on-insulator structure by water bonding of the plates on thermally oxidized Si substrates, thus permitting a further thinning of the diamond plate to <500 nm by Ar/Cl plasma reactive-ion-etching (RIE). The cantilevers are then patterned by standard optical lithography and released by substrate etching. A similar wafer bonding–based technique has also been used to form a diamond/silica/silicon multilayer structure^39^. The other method consisted of clamping the diamond plates to two SiO_2_ substrate central apertures. This provides a diamond silica sandwich for optical lithography on one side and thinning on the other side, resulting in a residual 5 μm diamond ledge with reduced clamping losses at the base of the cantilevers (Figure 1a).

Another technique to fabricate free standing mechanical and photonics nanostructures is the so called angle etching technique^26^. It is based on electron-beam-lithography (EBL) and anisotropic plasma etching in two steps, the second step being at an oblique angle to the sample surface. It consists of first patterning a 200 nm titanium etch mask by EBL; an oxygen-based plasma etching is first used to transfer the mask to the bulk diamond, which is etched down to 600 nm. A second anisotropic etching is then performed at an oblique angle to release the structure. This method is also used to fabricate free-standing nanoscale components in bulk single crystal diamond, including optical waveguides, PHC and microdisk cavities^26^.

**Figure 2:**
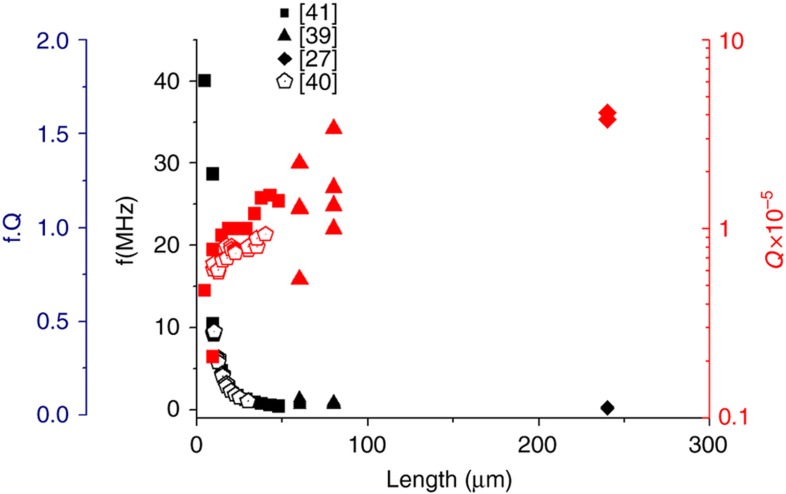
Data from Refs. 27,39–41 showing *f*, *Q*_m_ and *f*_c_·*Q*_m_ versus the length for single clamped nanobeams single crystal diamond measured at room temperature in vacuum.

Single and double clamped cantilevers were fabricated with rectangular or triangular cross section. For triangular cross section cantilevers fabricated with the ‘angle etching’ method, the nano-beam thickness, *t*, is linked to the width, *w*, via the relationship^40^

$$t=\frac{w}{2\tan\theta }$$

where ≈50°. For rectangular singly nanobeams, the measured frequencies modeled by $f_{c}=0.162\frac{t}{l^{2}}\sqrt{\frac{E}{\rho }}$ (Figure 2) experimentally follow a *f*_c_∝*l*^−2^ scaling law.

For double clamped cantilevers, the frequency dependence on the length is not monotonic due to uniaxial compressive stress along the length of the beam *σ*. Thus, for a rectangular doubly clamped cantilever, the vacuum frequency will be reduced by the term under square root^42^

$$f_{c}=1.03\frac{w}{l^{2}}\sqrt{\frac{E}{\rho }}\times \sqrt{1+\frac{\sigma l^{2}}{3.4Ew^{2}}}$$

For doubly clumped triangular cross sectional cantilevers, a similar behavior to the in plane and out-of-plane frequencies occurs depending on *σ* (Ref. 40).

In Table 1 we summarize the most relevant results on the single clamped cantilevers dimensions, for a given frequency and *Q*_m_ compared at room temperature and vacuum conditions for electronic-grade single crystal diamond^27,38,41^ and optical-grade single crystal diamond^40^. Figure 2 shows these data for clarity. The geometry of the cantilever, mostly thickness and length, introduces the major variability on *Q*_m_ and *f*_c_.

In Ref. 39 cantilevers were realized with lengths *l*=60 and 80 μm, *w*=15 and 20 μm and with varying thickness *t*=0.76/2.1 μm. Room temperature *Q*_m_=338 000 at *f*_c_=730 kHz (size 80×20×1.5 μm) was obtained while the maximum room temperature^27^ *Q*_m_=1 200 000 for *l*=800 nm in electronic-grade single crystal diamond, with *Q*_m_≈5.8×10^6^ at *f*_c_=32 kHz in the millikelvin regime.

In Ref. 40, free-standing single crystal diamond singly and doubly clamped diamond nanobeams with triangular cross section were fabricated, both by the angled-etching methodology^26^.

Compared to the singly clumped cantilevers of the same length and width where the axial stress is released by the free-ended structure, the doubly clumped cantilevers provide a lower frequency and a lower *Q*_m_. For example, a 675 nm wide, 55 μm long doubly clamped diamond nano-beam shows a maximum *Q*_m_~19 000 for 6.1 MHz at room-temperature in a high vacuum, showing a discrepancy from Euler-Bernoulli theory, which strongly indicates a compressive stress due to the fabrication process.

In Ref. 27, nano-cantilevers with thickness *t*=80–800 nm, *l*=20–240 μm and *w*=8–16 μm were realized and in Ref. 41 the nanobeams realized were fixed at a height of *h*=530 nm, *w*=820 nm, 1.3 μm and *l*=4.8–48 μm, the dimensions *w* and *l* being comparable to the other methods. *Q*_m_ increases for longer devices due to reduced clamping losses, while *f*_c_ reduces for longer cantilevers.

The temperature effects on the cantilevers and their thickness are the major reasons for the large variability of *Q*_m_ and *f*_c_ due to mechanical dissipation. It has been observed that *Q*_m_∝*t*, thus indicating the presence of surface friction. Surface friction is reduced at cryogenic temperatures, providing the higher *Q*_m_. Surface friction mechanisms were tested^27^ by modifying the cantilever’s surface species by oxygen and fluorine termination on both optical-grade and electronic-grade single crystal diamond cantilevers. In the first case, an improvement factor of 10 for *Q*_m_ was observed for the same temperature and thickness, indicating that specific atomic species layers on the surface can reduce/increase surface friction.

**Figure 3:**
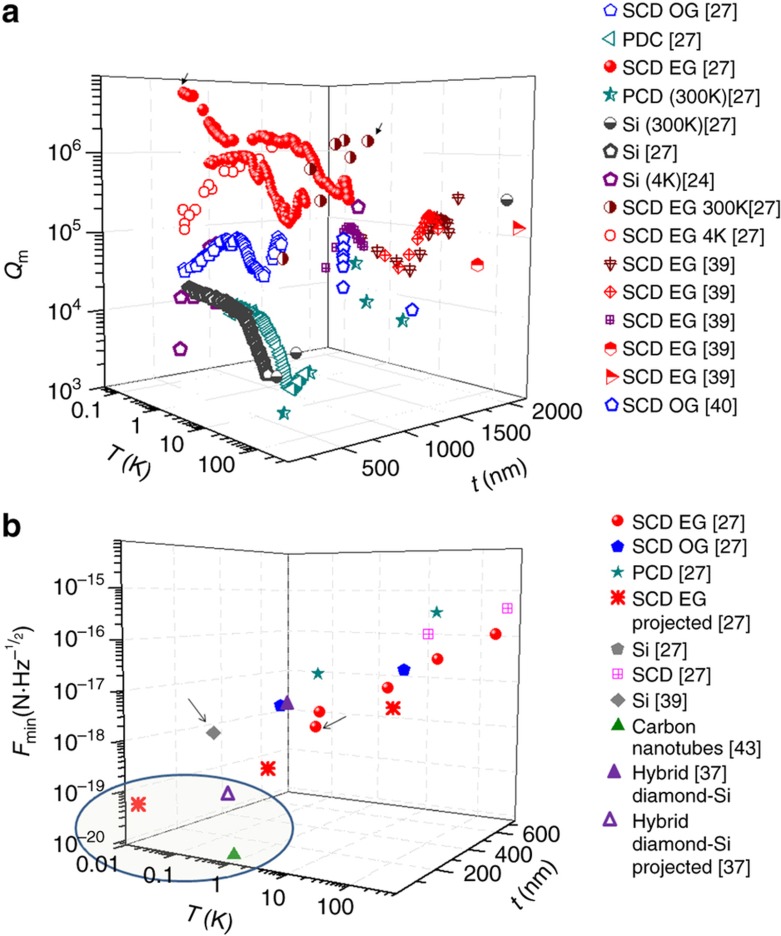
(**a**) Measured *Q*_m_ values for different temperatures, cantilever lengths and materials: single crystal diamond (SCD) in electronic-grade (EG) and optical-grade (OG), polycrystalline diamond (PCD) and single-crystal Silicon (Si). Arrows indicate record *Q*_m_ reported in the text. (**b**) Inferred and projected sensitivities for force measurement of different cantilevers versus temperature and thickness for *B*=1 Hz. Data from Refs. 24,27,37,39,40,43. The arrows indicate current best realization with diamond and silicon, while the circles indicate the required sensitivity for nuclear magnetic resonance signals using spin magnetic resonance force microscopy. Experiments in the range 3 K to 300 K show^27^ that EG SCD gives *Q*_m_ factors up to one order of magnitude higher than optical-grade single crystal diamond, comparing cantilevers of similar thickness (100–300 nm).

Figure 3a shows a summary of *Q*_m_ measured values versus temperature and cantilevers thickness, for electronic-grade and optical-grade single crystal diamond. Polycrystalline diamond (PCD) and single crystal Si are also shown for comparison. This comparison determines the material role, temperature and thickness optimization to achieve the highest force sensitivity. The sensitivity in rectangular cantilevers is

$$F_{\min}=\sqrt{\frac{wt^{2}k_{B}TB}{lQ_{m}}\sqrt{E\rho }}=\sqrt{\frac{wk_{B}TB}{l}\alpha },$$

where $\alpha =\frac{t\sqrt{E\rho }}{Q_{m}}$ is related to the mechanical dissipation as discussed in Ref. 27.

It has been inferred by determining the noise temperature of the cantilevers from their measured displacements $T=\frac{k_{c}x^{2}}{k_{B}}$ and using measured values of *f*_c_, *Q*_m_, *k*_c_.

In Figure 3b, we show a summary of the force sensitivity inferred and projected.

At present, diamond nanocantilevers’ best sensitivity has been measured to be in the order of 0.54 aN Hz^−1/2^ at a temperature of 100 mK for a 660-nm thick cantilever^27^. This is already better than a single-crystal Si of 290 nm thickness at 220 mK and roughly the same material purity (0.82 aN Hz^−1/2^)^43^. The projected limit of the technology based on these recent works (240·1·0.05 μm) would bring the thermal noise floor to the 10–100 zN Hz^−1/2^ range^27^. The thinnest built 60·15·0.75 μm cantilever in Ref. 39 shows a best sensitivity of 0.4 fN Hz^−1/2^ at room temperature and 0.1 fN Hz^−1/2^ at 10 K. In Ref. 37 a sensitivity of 2.4 aN Hz^−1/2^ at 4 K is achieved using a diamond nanowire integrated with an ultrahigh sensitivity Si cantilever and a projected sensitivity of 0.65 aN Hz^−1/2^ at 300 mK.

The nanocantilever dynamics for applications, such as mass sensing, magnetometry and scanning probe microscopy, needs to be measured in nanoscale fluids, as biochemical analysis of samples is usually performed in ambient air or liquids. Ref. 41 measured the pressure-dependent mechanical dissipation $1/Q_{m}^{g,l}$ of the triangular cross section singly clumped nanobeams, operating in light (He) and heavy gases (N_2_ and Ar) $\left(1/Q_{m}^{g}\;\right)$ and water $\left(1/Q_{m}^{l}\right)$. The *Q*_m_ and frequency were measured in vacuum conditions and compared to the gas and fluid conditions to determine the fluidic dissipation.

For a cantilever of 29·0.820·0.530 μm^3^, as reported in Table 1, by going from vacuum to gas, the resonance frequency does not shift significantly (*f*_g_=1.208 MHz) while the dissipation increases, $Q_{m}^{g}=100$; in water both frequency and dissipations change significantly, *f*_w_=0.43 MHz and $Q_{m}^{w}=1.1$. The interaction of the gas with the cantilever was studied, but changing the pressure *p*, $1/Q_{m}^{g}\propto p$ for low pressure (*p*<*p*_c_ in the condition of molecular flow, that is, the gas molecules interact only with the surface of the cantilever), while $1/Q_{m}^{g}\propto \sqrt{p}$ (*p*>*p*_c_, indicating the transition to viscous flow). It has been observed that *p*_c_ depends linearly on the frequency for high-frequency cantilevers (*f*_c_>10 MHz). The wider cantilevers with the same length, and hence the same frequency, provide a lower *p*_c_ at which the transition occurs. In the low-frequency regime, the transition from molecular flow to viscous flow occurs when *w*~*λ*, where *λ* is the mean free-path of the gas molecules. When using the same cantilever in different gases (He and Ar), *p*_c_ is lower for Ar due to its larger molecular diameter.

### Diamond nanowires

In scanning probe microscopy, the force sensitivities deteriorate as the tip becomes close to a surface due to noncontact friction effects, depending on dielectric fluctuations^44^, radiative heat transfer and the presence of static or fluctuating surface charge^37^. Apart from the use of Si nano-wires^39^, nano-mechanical detection of ultrasmall forces was achieved with freely vibrating nanobeams or particles that were ≳100 nm from a surface.

Diamond due to a low dielectric constant has the potential for low noncontact friction. In Ref. 37 diamond nanowires of a few micrometers long and diameters of around 100 nm were fabricated using two different masking schemes and applying masked etching of single crystal substrates by inductively coupled plasma reactive ion etching. One scheme was based on micromachining of received diamond surface defects acting as intrinsic masking, the other by deterministic fabrication of diamond nanowires using an electron beam–defined alumina etch mask. The pillar geometry is made of a pyramidal base connected to a nanowire-like tip via a thin waist of ~10 nm (Figure 1 i–ii).

The diamond nanotips were integrated with a Si cantilever with a sensitivity of a few aN·Hz^−1/2^, *Q*_m_≈130 000 and *f*_c_≈7.6 kHz at 4 K. The cantilever tip *f*_c_ and *Q*_m_ reduced only when the diamond tip was ≤10 nm from a gold surface (*Q*_m_≈40 000 at 1 nm from the surface). The values of noncontact friction were estimated to be comparable with high-frequency (1 MHz) Si nanowire resonators^45^.

In force detected MRI methods, a sample should be positioned as close as possible to a nanoscale ferromagnetic tip, generating a strong magnetic force on a spin, which determines the spatial imaging resolution. At ≲10 nm a magnetic force gradient of 150 Gauss/nm can be achieved, creating a force on a single proton spin of ≈0.21 aN. This force value corresponds to a diamond nanowire tip cantilever sensitivity at 10 nm distance and at 4 K of 2.4 aN Hz^−1/2^, while at 300 mK, the sensitivity of the diamond nanowire tip cantilever can be 0.65 aN Hz^−1/2^.

### Diamond dome resonators

The first nanomechanical resonators micro fabricated in single-crystal diamond were dome-type resonators (Figure 1h) built with a 70-nm thick shell^36^ (the current thinner single crystal diamond cantilevers were 76 nm thick), demonstrating a quality factor *Q*_m_≈4000 at room temperature (*f*≈27 MHz), increasing to *Q*_m_≈20 000 at 10 K and covering a wide range of resonant frequencies from 10 to 600 MHz. Here the main loss mechanisms were introduced by the fabrication process, based on C^+^ ion implantation to create a loss sacrificial layer. In addition, a high N_s_ concentration substrate was used.

The resonance frequency of this resonator is modeled as a clamped circular plate^46^ whose mechanical frequency depends linearly on the height and inversely on the square of the radius of the plate. Energy dissipation in single crystal diamond annular plate resonators (175 nm thickness) has been measured for temperatures ranging from 4 to 300 K at resonant frequencies around 50–55 MHz (Ref. 46). An order of magnitude reduction in dissipation is observed as the temperature is lowered from room temperature (1/*Q*_m_=5×10^−4^) to 30 K (1/*Q*_m_=5×10^−5^).

## Nanophotonics components

Nanophotonics is an interface between optical elements, such as optical fibers and lenses used to transfer optical signals, and solid-state physical objects, such as currents and spins in a material, color centers and nanomechanical objects^47^. Diamond also provides the opportunity to harness quantum nanophotonics when photons are used to interconnect quantum systems such as spin states associated with luminescent individual color centers in diamond, such as NV-centers. A recent review with a focus on quantum nanophotonics and the integration of color centers in diamond can be found in Ref. 48. These color centers can constitute quantum bits of information and their readout occurs by the observation of a single-photon emission associated with the manipulation of their electron spin state and of the nearby coupled nuclear spin sublevels. To make these and other applications viable, the efficiency of information exchanged between the NV-center’s electron spin and a photon is of central importance. While the realization of nanophotonic components in diamond has been an active area of research for almost a decade^1^, in this review, only the latest nano-photonic components achieved in single crystal diamond are considered. We focused on broadband photon collection efficiency enhancement components such as diamond nanopillars, narrow bandwidth devices such as disk resonators, ring resonators and one-dimensional (1D) and two-dimensional (2D) PHC cavities. The latter components are characterized by their optical *Q*-factor ~*λ*/Δ*λ* and mode volume *V*~(*λ*/*n*)^3^. Nano-cavities can be used to modify the spontaneous emission (SE) of color centers in the diamond^49^. To increase the possibility to use NV-centers in quantum networks or in magnetic sensing more efficiently, it is necessary to force the SE to be mostly in the Zero Phonon Line (ZPL; naturally only 3% of its photoluminescence is in the ZPL). To achieve this, it is necessary to couple the SE with one optical cavity mode to generate a Purcell enhancement

$$F_{p,\max}=\frac{3}{4\pi^{2}}{\left(\frac{\lambda_{\mathrm{cav}}}{n}\right)}^{3}\frac{Q}{V}$$

while the SE enhancement is given by 50,51,52,

$$F_{\mathrm{SE}}=\xi F_{p,\max}\frac{1}{1+4Q^{2}{\left(\frac{\lambda_{\mathrm{ZPL}}}{\lambda_{\mathrm{cav}}}-1\right)}^{2}}$$

where *ξ* accounts for the spatial and angular overlap between the emitter dipole moment and the cavity mode intensity. Typically, $F_{\mathrm{SE}}\ll F_{p,\;\max}$, even if the theoretical value can differ from the actual measured *F*_SE_. While the total fraction of emission into the ZPL once in the cavity is given by *ξ*_ZPL_*F*_p, max_, where *ξ*_ZPL_ is the fraction of emission of the defect in the ZPL in the bulk material, about 3% for NV center.

However, since not all the optical resonators are coupled with a specific color center, we compared them with respect to the measured *Q* and theoretical *F*_p, max_ based on the cavity designs. PHC cavities are characterized by high *F*_p, max_ due to a smaller mode volume. The design of the cavities requires optimization of *F*_p, max_ while the integration of the center in the cavity requires the optimization of *F*_SE_ to achieve maximum SE enhancement.

**Figure 4:**
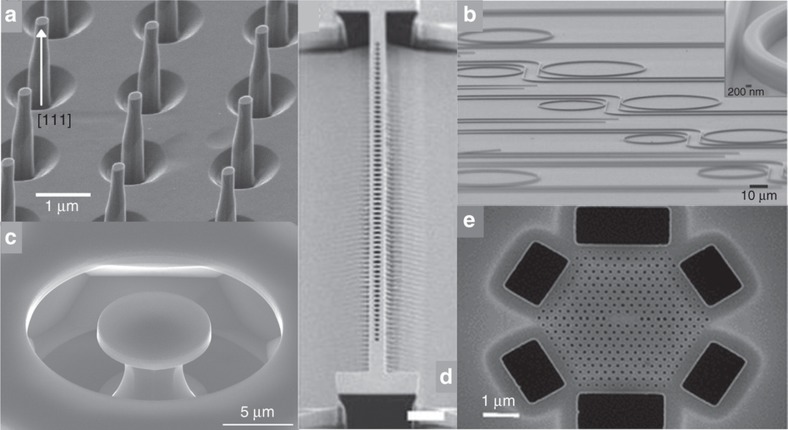
Scanning electron microscopy images of: (**a**) Diamond nanopillars fabricated on a (111)-oriented single-crystalline diamond sample. Image reprinted from Ref. 53 with the permission of AIP Publishing. (**b**) An array of waveguide-coupled single crystal diamond ring resonators on a SiO_2_/Si substrate obtained by the thin-down and electron beam lithography (EBL) techniques. The rings are 850 nm high and 875 nm wide and have radii of 20–30 μm with record *Q*-factor~10^6^ at λ=1545 nm. Images reprinted by permission from Macmillan Publishers Ltd: Nature Photonics. Ref. 54, copyright 2014. (**c**) 7.9 μm diameter microdisk in a diamond chip with 〈100〉-oriented surface and edge crystal planes fabricated by undercut and EBL techniques, *Q*~115 000 at *λ* =1552 nm. Image reprinted (adapted) with permission from Ref. 55. Copyright 2015 American Chemical Society. (**d**) 1D nano-beam cavity with width ~1 μm, fabricated by angle-etching method and EBL techniques, resonant at *λ*=1, 680 nm, made of elliptical holes with lattice constant ~536 nm and radius of the holes ~146 nm. The record loaded *Q*~183000. Image reprinted by permission from Macmillan Publishers Ltd: Nature Communications: Ref. 10, copyright 2014. (**e**) Linear three-hole defects 2D-PHC cavity fabricated in a triangular lattice with period a~218 nm, hole radius *r*=0.29·a~63 nm, and membrane thickness *h*=0.91·a~198 nm, optical *Q*-factor~3000 and *λ*=637 nm. Fabricated by thin-down technique using reactive ion etching and EBL. Image reprinted figure with permission from: Ref. 50. Copyright 2012 by the American Physical Society.

In Figure 4, we show scanning electron microscopic images and details of nanophotonics components fabricated in single crystal diamond^10,50,53–55^. Among the optical resonators described in the following sections, we show those currently achieving the highest *Q*_s_ at specific wavelengths in the near infrared and in the visible spectrum at around 637 nm. This is in the ZPL of the NV center, or in the spectral region 600–750 nm, where known color centers^56^ in diamond with single photon emission can be integrated.

**Figure 5:**
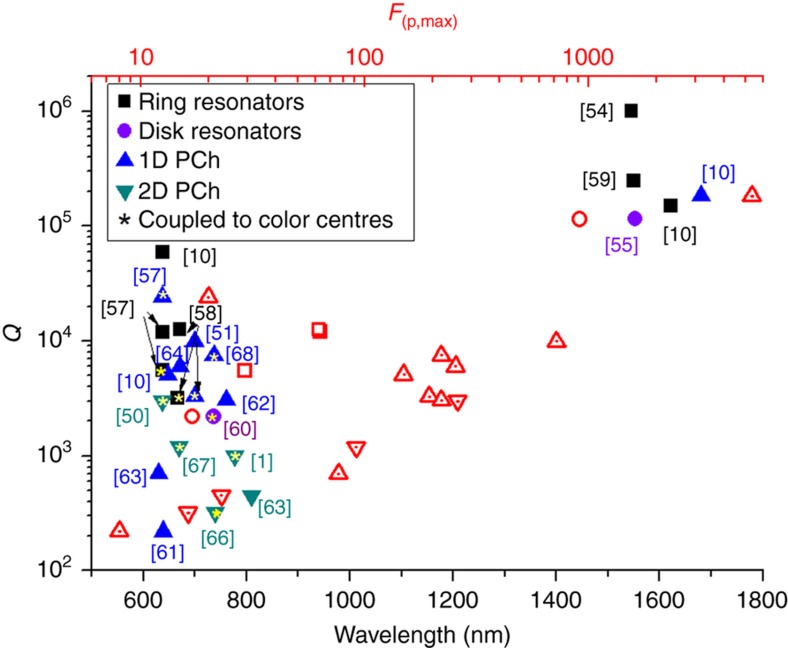
Optical *Q*-factors measured for different optical resonators and expected maximum enhancement based on the mode volume design *F*_p, max_ he cavity coupled with color centres are indicated with star. Data from Refs. 1,10,50,51,54,55,57–68. We observe that the experimental *Q*-factors generated when the same cavity is coupled to a color center tend to be much smaller compared to an unloaded cavity and the *F*_SE_ tends to be much smaller compared to the expected *F*_p, max_. By way of an example in Ref. 50 a measured loaded *Q*-factor of 3000 (theoretical *Q*=6000) is achieved with a record *F*_SE_=70, while the expected *F*_p, max_~260 based on the cavity mode volume. In Ref. 51 *F*_SE_=63 while the expected *F*_p, max_~194.

In Figure 5, data from Ref. 1,10,50,51,54,55,57,58,59,60,61,62,63,64,65,66,67,68, we summarize the measured *Q*_s_ and the predicted *F*_p,max_ enhancement at specific wavelengths corresponding to ZPLs color centers in diamond (visible) and for typical infrared resonance modes. It is observed that higher *Q*_s_ can be obtained in the near-infrared due to larger cavity dimensions less limited by the fabrication techniques and in larger resonators, such as ring resonators. Among the PHC cavities, the 1D-PHC cavities have achieved the highest observed Q-factors and *F*_p, max_ due to smaller mode volume, compared to the same parameters achieved in 2D-PHC cavities. Thus, the fabrication procedures are still a major hurdle in the achievement of the expected Purcell enhancement as well as the placement of the color center at the exact locations within the maximum intensity of the cavity modes volume.

**Figure 6:**
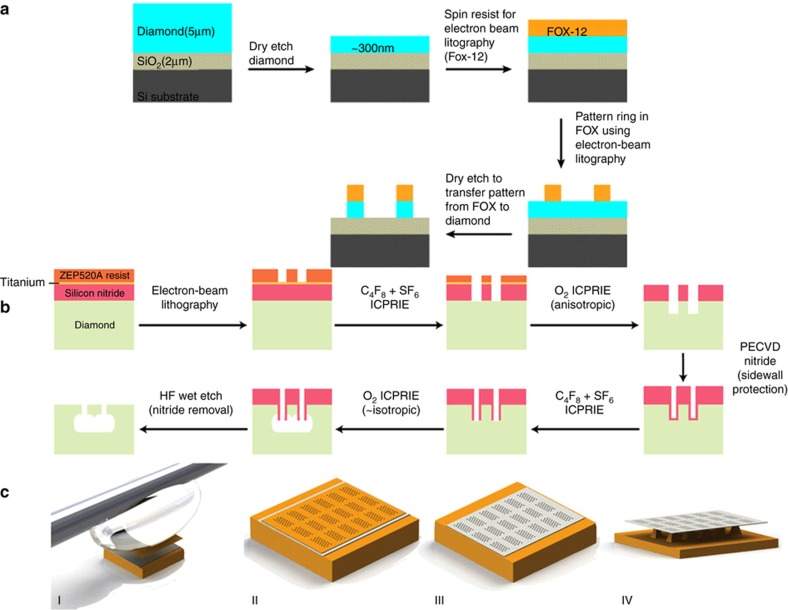
Examples of diamond nanofabrication and patterning. (**a**) Dry etching (oxygen plasma in an Oxford RIE etching machine) thin-down technique of 5 μm-thick diamond membrane until it is 300 nm thick placed on a 2 μm-thick SiO_2_ layer grown on a silicon wafer. The electron-beam resist (Fox12) is spun on the chip and electron-beam lithography used to pattern a ring resonator. The pattern is transferred from the resist to the diamond using dry etching in an oxygen plasma. Image available under the terms of the Creative Commons Attribution 3.0 License from Ref. 57. (**b**) Process for fabrication of diamond nanobeams using quasi-isotropic reactive-ion undercut etching. Image available under the terms of the Creative Commons Attribution 3.0 License from Ref. 32. (**c**) Patterning of a diamond membrane using a silicon membrane as a contact etch mask. (I) Transferring of a Si mask onto a <300 nm diamond membrane using a micro PDMS adhesive. (II) The silicon membrane on top of the diamond membrane is used as an etch mask for oxygen plasma etching. (III) The diamond membrane is patterned during oxygen etching with subsequent mask removal. (IV) A SF6 isotropic dry etching removed the silicon underneath and suspended the diamond membrane. Image reprinted by permission from Macmillan Publishers Ltd: [Scientific Reports]: Ref. 69, copyright 2015.

The fabrication of photonic components in single crystal diamond for nanomechanical resonators suffers from the fact that a thin film or a freestanding diamond membrane of 200 nm is necessary for photonic devices. The adopted methods to achieve a diamond membrane of 200 nm or less are common to the fabrication of nanocantilevers and they can be grouped under the following methods (Figure 6, images from Refs. 32,^57^,^69^):

- The ‘thin-down’ method, where a diamond substrate with a thickness of tens of microns from commercial suppliers is thinned down by dry etching^52,57^ (Figure 6a), or RIE, to a thickness of some hundreds of nanometers^59^.
- The ‘lift-off’ technique^65,70^, which consists of creating a sacrificial layer by irradiating the diamond with ions. This layer is removed after graphitizing it by annealing. A regrowth of a pristine diamond layer is performed to remove any left diamond damaged and the thin layer is then transferred to a different substrate to create nano-structures^71^.
- The ‘angle-etching’ technique, described earlier^10^.
- The ‘undercut’ etching method, where a resist is patterned by EBL and transferred on the bulk diamond; a final undercut of the structures being required to achieve freestanding devices on top of the bulk diamond. The undercut typically introduces damage to the devices. A process flow for creating diamond nano-beams using quasi-isotropic reactive ion undercut etching is shown in Ref. 72
- Quasi-isotropic oxygen undercut, based on conventional vertical RIE and oxygen plasma etching at elevated sample temperature^12,32,55^ (Figure 6b).
- Focused ion beam (FIB) milling typically uses Ga or O_2_ ions to directly cut the diamond without the need for generating a mask. Ref. 61 uses for example Ga FIB to fabricate nanostructures. Its main drawbacks are related to damage due to implanted ions and lack of scalability of the process due to its long milling time^73^.

A new approach to diamond nanofabrication has been recently proposed in Ref. 74. They employed reactive ion beam angled etching (RIBE) to manufacture optical resonators. They report *Q* of ≈30 000 in bulk polycrystalline and ≈286 000 in single crystal diamond. The technique may have advantages over comparable techniques to produce freestanding nanostructures with better scalability.

For nanopatterning of the cavities, the following methods are used: (a) FIB milling defines and transfers the photonics pattern directly in the diamond but its accuracy is limited to 10 nm in addition to introducing damage to the diamond^61^; (b) EBL defines a pattern in an electron beam resist layer and dry etching is used to transfer the photonic structures into the diamond^64^; (c) Si mask lithography has also been used due to the relatively mature fabrication process of silicon-on-insulator (SOI), where a Si mask is patterned on an Si insulator that is released and transferred to a diamond membrane, the diamond membrane then being patterned using oxygen RIE^62,69^.

### Diamond nano-pillars

Diamond nano-pillars and nano-wires as nano-photonics component are used to increase the collection efficiency of photons emitted from an NV or can be used as scanning probes for magnetometry and diamond cantilevers for force-sensing.

The first single crystal diamond nano-wire^75^ was 2 μm tall and 200 nm wide, acting as a waveguide in a dielectric antenna, with a single NV center photons collection enhancement factor of 10. Similar enhancements can be achieved by using solid immersion lenses etched into the diamond surface^76^. The fabrication is scalable as nanowires can be fabricated in parallel on the same chip^77^. One of the limitations is due to the fabrication of nano-pillars on a (100) facet single crystal diamond as ideally, an NV dipole should be perpendicular to the pillar’s axis for maximum collection efficiencies^78^, while it forms an angle with the pillars, axis due to the NV-center orientation aligning along one of the four equivalent <111> crystal-directions. As high-quality, high-purity (111)-oriented chemical vapor deposition diamond has become available, Ref. 53 demonstrated the fabrication of single crystal diamond nano-pillars on this (111)-oriented substrate. This crystal orientation provides a factor of 2–3 further enhancement of the collection efficiency compared to previous demonstrations.

### Ring resonators

Ring resonators are photonic elements etched into single diamond-on-insulator (a SiO_2_ substrate) or free standing (suspended over air) diamond thin films. Ring resonators are generally coupled to waveguides and are relevant for complex architectures enabling on-chip photonic functionalities as opposed to stand-alone components.

Ref. 58 integrated a ring resonator coupled to an optical waveguide with grating in- and out-couplers to direct a NV-center inside the ring resonator as a source of photons to the waveguide output. Ring resonators have high quality factors of *Q*=12 600, while *Q*=3200 when NV-center single photons in the resonator are coupled out of the waveguide. Similarly, Ref. 57 fabricated a microring resonator coupled to a ridge waveguide with similar uncoupled and coupled *Q*-factors, providing a measured *F*_SE_=12 at 637 nm.

Ref. 59 developed a high-quality factor single crystal diamond race-track resonator, operating at near-infrared wavelengths (1550 nm) using a very similar fabrication method with *Q*~250 000.

In contrast to the above methods, in Ref. 10 suspended racetrack resonators and a nanobeam photonics crystal cavity was realized from bulk diamond using the angle etching technique with *Q*=151 000 at 1622 nm. Single crystal diamond ultrahigh-quality-factor (10^6^) diamond ring resonators operating at telecom wavelengths were fabricated^54^, used as a diamond non-linear photonics platform where optical parametric oscillations were observed via a four-wave mixing.

### Microdisks

Microdisk resonators sustain ‘whispering gallery’ electromagnetic modes and they are typically stand-alone devices where the emission is out-coupled using evanescent fiber couplings. Hence, they have limited applications in integrated nanophotonics. Disk resonators are mostly considered due to the simplicity of fabrication. They were the first optical cavity type studied in diamond. Ref. 60 fabricated single crystal diamond microdisks by thinning down a diamond membrane with further epitaxial overgrowth of a thin single crystal diamond film. The microdisk was coupled with the ZPL of an ensemble of Si vacancy defects (SiV) with ZPL at 738 nm, a loaded *Q*=2200, a very moderate FSE of 1.3 and a mode volume *V*~9.6·(*λ*/*n*)^3^. Ref. 55 developed microdisks supporting TE- and TM-like optical modes with *Q*>1.1·10^5^ and V<11·(*λ*/*n*)^3^ at a wavelength of 1.5 μm.

### PHC cavities

The realization of interconnected high-*Q* cavities for quantum information technologies should be in the form of a 1D nano-beam or 2D-slab PHC. In fact, these architectures could permit to produce large scale systems with multiple emitters per cavity strongly coupled as well as arrays of multiple emitter-cavity nodes to implement entanglement generation, quantum memories and photonic or spin qubits quantum gates. An example of entangling two SiV centers strongly coupled in diamond nano-cavities is shown in Ref. 68, as a first tangible step in this direction. Similarly, efficient coupling of germanium vacancy in diamond waveguides has been achieved, demonstrating non-linear properties at the single photon^9^.

The first 1D nano-beam was fabricated in diamond using FIB^61^. The holes’ distance defines a lattice constant *a*=205 nm for visible modes confinement with very low quality factors of *Q*=220. A triangular nano-beam PHC cavity by varying the crystal lattice constant a0-a=220÷240 nm with predicted *Q*~2×10^6^, was fabricated by Ref. 62, yielding a measured *Q*~3000. 1D-PHC cavities were fabricated with a lattice constant *a*=200 nm and a hole size deterministically varying within the nano-beam, achieving a small *V*~0.7ˑ(*λ*/*n*)^3^ designed to be in resonance with the NV-center ZPL. The maximum observed quality factor was 700 and no coupling to color centers was observed for the 1D-PHC cavity^63^.

A 1D-PHC cavity^64^ consisting of a series of holes etched through a diamond ridge waveguide had *Q*=6000 and was demonstrated to couple the cavity modes of a single Nitrogen NV with *F*_SE_=7.

Nanobeam PHC cavities were fabricated both in the near IR and at 637 nm by adapting the angle-etching techniques^10^. These devices consist of a waveguide with elliptically shaped holes designed to have the highest *Q* and the smallest mode volumes. The fundamental resonance at *λ*~1, 680 nm has a *Q*~3×10^6^ and a *V*~2.26·(*λ*/*n*)^3^. For *λ*~637 nm the cavity parameters were scaled down with *Q*=33, 000/59, 000, while the best *Q*~8200 for the coupled waveguide cavity mode at 710 nm.

In addition to improving the cavities parameters, attention has been paid to deterministically incorporate NV in the diamond close to the cavity mode. PHC nano-beam cavities^65^ were coupled to the NV’s ensemble by incorporating a delta-doping technique, based on growing a nanometer-thick diamond nitrogen-doped layer. The cavities were fabricated with a linearly tapered lattice constant with a *Q*~24 000 and *V*~0.47·(λ/*n*)^3^. Regardless of such a small volume, the *F*_SE_=20 for the ZPL of the NVs ensemble, results much lower than expected.

**Figure 7:**
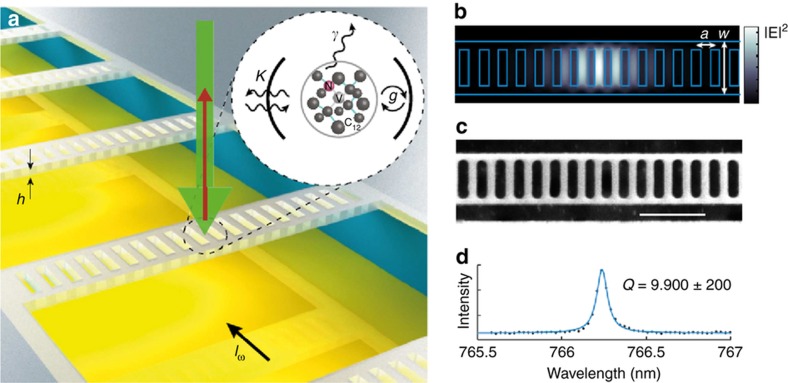
(**a**) 1D PhC cavities integrated on a Si substrate with metallic strip lines to provide microwave excitation for NV-center coherent spin control. The cavity is a rectangular nanobeam based on a suspended 1D diamond PHC structure with a lattice constant *a*=220 nm, beam width *W*=2.4·a and a thickness of *h*=0.7·a. The inset shows the NV-nano cavity system with the NV-nano cavity coupling *g*, *γ* is the NV natural SE decay rate and *k* is the cavity intensity decay rate. The defect was positioned in the cavity by ^15^N-ions implantation and subsequent annealing at 850 °C. (**b**) Simulated electric field intensity for the optimized fundamental cavity mode showing high confinement at the center of the cavity. (**c**) Scanning electron microscope (SEM) of a representative cavity structure. The scale bar represents 1 μm. (**d**) Measured cavity resonance with a quality factor *Q*~9, 900±200. Images reprinted by permission from Macmillan Publishers Ltd: Nature Communications: Ref. 51, copyright 2015.

In Ref. 51 it is reported that the highest *F*_SE_=62 is achieved for a single NV in a 1D-PHC cavity with a measured *Q*~9900 and *V*~1.05·(λ/*n*)^3^ (Figure 7). A linear increase of the lattice constant from 0.9·*a* to *a* in increments of 0.02·*a* per period away from the center, defines the cavity defect state. When strongly coupled to a single NV, the cavity *Q*~3300.

Recently a breakthrough in integrated diamond nano-photonics has been achieved by integrating SiV in a 1D-PHC cavity with *V*~2.5 (*λ*/*n*)^3^ and *Q*~7200, by implanting Si ions at the center of the cavity to create the color centers. The strong coupling of single SiV with the cavity permitted to observe optical nonlinearity at the single photon level^68^.

2D-PHC cavities were fabricated in PCD, initially, with very low *Q*-factors^1,4,48^. An experimental *Q*~1000 and a current *F*_SE_=70 related to the coupling of the cavity with a single NV-center, was achieved in Ref. 50. The cavity is a linear three-hole defect PHC cavity fabricated in a triangular lattice with a period of *a*≈218 nm. Theoretical quality factors as high as *Q*~6000 can be achieved with this design with very small *V*~0.88·(*λ*/*n*)^3^.

Ref. 63 develops 2D-PHC cavities with triangular lattice of air holes with lattice constant *a*=275 nm and *V*=1.5·(*λ*/*n*)^3^, coupled, for the first time, to an ensemble of SiV centers. In Ref. 65 a 2D-PHC cavity with a triangular lattice of air holes and a one-hole type of defect was fabricated with a measured *Q* ~1000.

The deterministic coupling to a single SiV center was recently observed in a triangular lattice of air holes with a crystal lattice *a* ≈283 nm (Ref. 66). The nanocavity is formed by a linear one or seven holes’ defect in the photonic lattice. The fabrication of the cavity procedure was like the one reported in Ref. 63; however, the cavity was fabricated around a preselected single SiV color center in the membrane. This achieved a *Q*~320 once the cavity was coupled for a defect *F*_SE_=19, while the theoretical *V*~1.7·(*λ*/*n*)^3^.

A cavity was coupled with a single NV deterministically implanted at the center of the cavities^64^ achieving a maximum *Q* ~1200 and *V*~1·(*λ*/*n*)^3^. The cavity was fabricated with the same procedure as described in Ref. 66; however, only a modest *F*_SE_ was observed.

**Figure 8:**
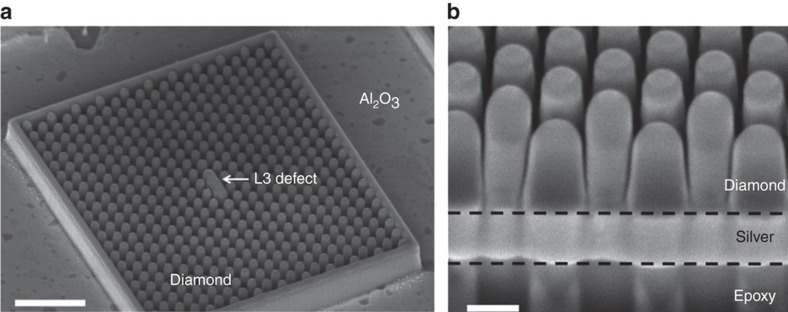
(**a**) Scanning electron microscope (SEM) micrograph of the diamond cavity (scale bar corresponds to 1 μm). (**b**) Focused ion beam (FIB) cross-section of a photonic crystal region (scale bar corresponds to 200 nm). The diamond-nanopillars are arranged in a hexagonal lattice, with a center-to-center spacing, *a*. The center of the lattice contains a linear defect, made up of three conjoined neighboring rods from the lattice, each with a radius of *R*, such that the length of the cavity is *L*=2·(*R*+a). The images are reprinted (adapted) with permission from: Ref. 80. 2015 American Chemical Society.

For application in magnetometry^79^ NV-centers should be placed as close as possible to the diamond surface, while the above-described high-*Q* cavities have their field maxima located centrally within the cavity. Hybrid metal-diamond PHC regardless of a smaller *Q* could provide better field localization. A hybrid metal-diamond PHC^80^ was fabricated using silver as the substrate and single-crystal diamond nano-pillars arranged in a hexagonal lattice with a cavity defect. The diamond nano-pillars are on top of a silver substrate, with a 5-nm layer of Al_2_O_3_ (*n*=1.8) sandwiched in between as the gap dielectric (Figure 8).

This hybrid cavity, whose fabrication is very challenging, had a measured *Q*=170 with a simulated V~0.1·(*λ*/*n*)^3^.

## Integrated nano-mechanical and optomechanics systems

There is currently a significant interest into the integration of nanomechanical components with the NV-center. An NV-center integrated in nanomechanical resonators could enhance current nano-MRI in scanning probe microscopy^81^. In addition, the coupling of the mechanical modes of the resonators with NV phonons permits modification and/or control of the spin coherence of the defect. The spin-phonons, interaction can lead to a new tool for imaging strain at the nanoscale and for coherent information transfer and manipulation between spin and mechanical modes as phonon-induced spin-spin interactions^82^. In addition, the spin coherence time of the single NV-center, normally up to 2 ms (Ref. 13), in bulk diamond at room temperature but collapsing to a few microseconds in shallow NV-centers due to surface effects^83^, could be significantly enhanced to improve magnetic sensitivity.

Finally, the combination of nano-mechanical systems with optical nanocavities naturally leads to opto-mechanical systems that, enriched with color centers with quantum mechanical properties, provide a route to undertaking a fundamental study in the domain of quantum optomechanics.

### Integrated nano-mechanical systems

NV diamond probes for magnetic imaging scanning probe microscopy are an example of integrated nano-mechanical systems. Scanning probe magnetic imaging permits the mapping of magnetic fields at the nanoscale, and was demonstrated at room temperature compatible with living cells by using a single diamond NV-center spin as a magnetometer^84^. A functionalized nano-diamond with NV-center magnetic spin is placed on a regular atomic force microscopy (AFM) tip, which provides the scanning function.

Alternatively, a single NV-center was positioned at 10 nm from the end of a high-purity diamond nanopillar mounted on an AFM^85^. This method enhanced the spin coherence time to 75 μs, and the NV collection efficiency due to wave guiding with 25 nm imaging resolution of the magnetic domains and magnetic field sensitivity of 56 nT Hz^−1/2^.

In Ref. 81, the de-coherence of a NV-center a few tens of nanometers from the tip of a 200 nm diameter scanning nanopillar was employed to image the randomly fluctuating magnetic fields from paramagnetic impurities on an underlying diamond surface. A monolithic diamond pillar is a scanning probe based on a NV-center used to image the magnetic domains on a hard disk surface and the vortices in an iron pnictide superconductor with 30 K critical temperature^86^. At 6 K temperature, the probe achieved a sub-100 nm spatial resolution for 3 μT Hz^−1/2^ direct current (DC) field sensitivity.

**Figure 9:**
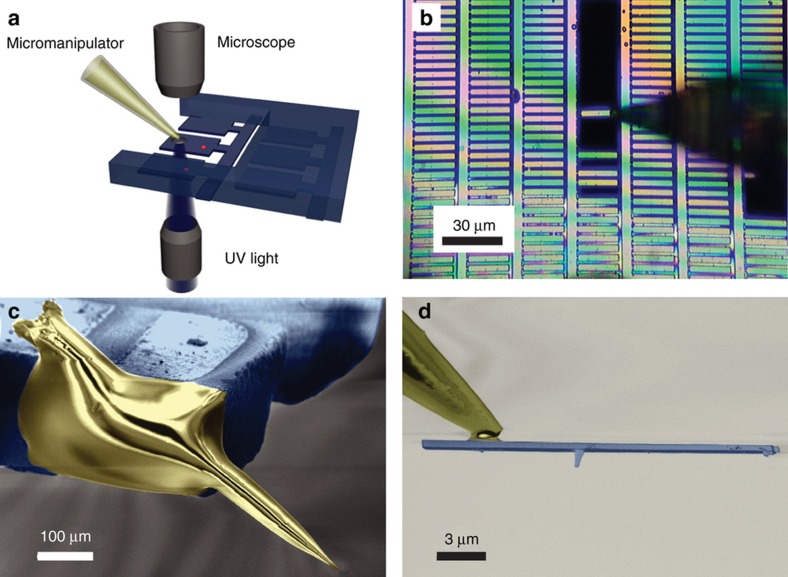
(**a**) The all-diamond scanning probe made of diamond cantilevers with diamond nanopillar are glued to a quartz tip. (**b**) Optical microscope image during the transfer process of the diamond probe to the atomic force microscopy (AFM) head. (**c**) Scanning electron microscope (SEM) image of the scanning probe attached to an AFM tuning fork. (**d**) SEM image of the final scanning probe attached to the end of the quartz tip. The cantilever was 20 μm long, 3 μm wide and connected to the diamond substrate via 500 nm bridges. The NV-center was created by ^14^N implantation at a depth of only 9 nm, reaching a trade-off with a spin coherence time of 76 μs (a shallow depth gives higher resolution, but also shorter coherence time due to proximal surface spins, and thus, lower sensitivity). Images reprinted from: Ref. 87, with the permission of AIP Publishing.

The magnetic stray field of single Ni nanorods was imaged with a resolution of 70 nm for scanning probes consisting of a scanning nanopillar (200 nm diameter, 1–2 μm length) on a diamond thin (<1 μm) cantilever structure (see Figure 9(d))^87^. The devices (diamond cantilever plus nano-pillar with integrated NV-center) with the best magnetic field sensitivity (50±20 nT·Hz^−1/2^ in an AC magnetic field and 200 nT·Hz^−1/2^ in a DC magnetic field) were transferred to an AFM head. The transfer of the scanning probe to the AFM tip is done using micromanipulators based on quartz micropipettes. The scanning probes were initially glued to the quartz tip using UV curable glue (Figure 9(a)), after a scanning probe was released from the substrate. Finally, the quartz tip carrying the scanning probe was glued to a tuning fork attached to an AFM head (Figures 9(c and d)).

**Figure 10:**
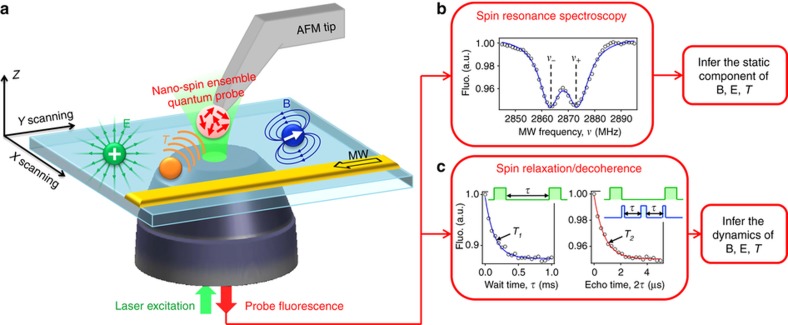
Schematic of the scanning nano-spin ensemble microscope. (**a**) Probe consists of a nano-diamond containing a small ensemble of electronic spins (symbolized by the red arrows) grafted onto the tip of an atomic force microscope (AFM). Optical excitation and readout, combined with microwave (MW) excitation, enable quantum measurements of the spin ensemble properties, such as spin resonance, relaxation and decoherence. Scanning the sample relative to the probe produces images of the sample with magnetic (B field), electric (E field) or temperature (*T*) contrasts, depending on the probing technique used. (**b**) Optically detected spin resonance spectrum of a nano-diamond on a tip. The solid line is the fit to a sum of two Lorentzian functions centered at frequencies *ν*±=*D*±*E*, providing the zero-field splitting parameters *D*=2868.3±0.1 MHz and *E*=5.4±0.1 MHz. (**c**) Spin relaxation (left) and spin decoherence (right) curve of a nano-diamond on the AFM tip. The inset depicts the sequence of laser (green) and MW (blue) pulses employed. Solid lines are fits to a single exponential decay, revealing a spin relaxation time of *T*_1_=142±9 μs and a spin coherence time of *T*_2_=780±30 ns. Images reprinted with permission from: Ref. 88, Copyright 2016 American Chemical Society.

The use of the AFM tip for magnetic scanning with single NV-centers usually brings about issues with the unavailability of simultaneous topographic scanning and the lengthening of the acquisition time. These were recently tackled by employing a nanospin ensemble of 100 NV-centers hosted in a nano-diamond (Figure 10), providing up to an order of magnitude gain in the signal-to-noise ratio (and thus acquisition time) with respect to a single NV-center, while preserving sub-100 nm spatial resolution^88^.

When integrated with nanomechanical systems, NV-centers show strong spin-phonon coupling in nanoscale dimension resonators^89–91^ mediated by the lattice strain effect as shown by incorporating photo-stable NV-centers in diamond cantilevers at different location from the cantilever base^90^. Experiments on NV-centers embedded at the base of 60 μm long, 15 μm wide and ~1 μm thick single-crystal diamond cantilevers^39^, where NV interacts with the fundamental mechanical mode of the cantilever via strain, permitted spin-based strain imaging with a sensitivity of 3·10^−6^ strain·Hz^−1/2^ (Ref. 90). The cantilevers have ~MHz mechanical frequency and *Q*_m_⩾3˙10^5^. The amplitude of the cantilever motion has been shown to impact the NV spin evolution. The strain susceptibility parameters parallel and perpendicular to the NV symmetry axis were determined from spin evolution measurements. Similar measurements were performed in Ref. 91.

To enhance the spin-phonon coupling strength, a smaller mechanical mode volume can be achieved by reducing the cantilever thickness and width to a few hundreds of nanometers while doubling its length. As demonstrated in Ref. 89 a single NV-phonon coupling rate of approximately 2 Hz was achieved. The cantilevers were fabricated using angle-etching technique^26^ with *w*=580 nm, *t=*170 nm and *l*=19 μm, *Q*_m_~10 000 and *f*_m_=937 kHz. It has also been shown that it is possible to control NV-center spins coherently by applying a stress in resonance with spin state splitting. This also permits access to spin transitions that are not accessible with AC magnetic field control^92,93^.

Recently, coherent control of the NV-center triplet state was shown in high-overtone bulk acoustic resonators (HBARs) by high-frequency mechanical stress resonators with a split frequency^92,93^. This parity nonconserving process enables access to forbidden transitions by common optical and microwave spin sequence excitations.

Internal time-varying strain fields in diamond cantilevers can also induce coherent oscillations in an embedded NV-center, reaching the strong-driving regime at room temperature and enhancing the spin coherence time^94^. In fact, diamond nanocantilevers coupled with NV-centers gain access to the strong spin-phonon coupling regime, where the mechanical dynamics can be detected by electron spin resonance and spin echo measurements in the time domain.

### Integrated optomechanics

**Figure 11:**
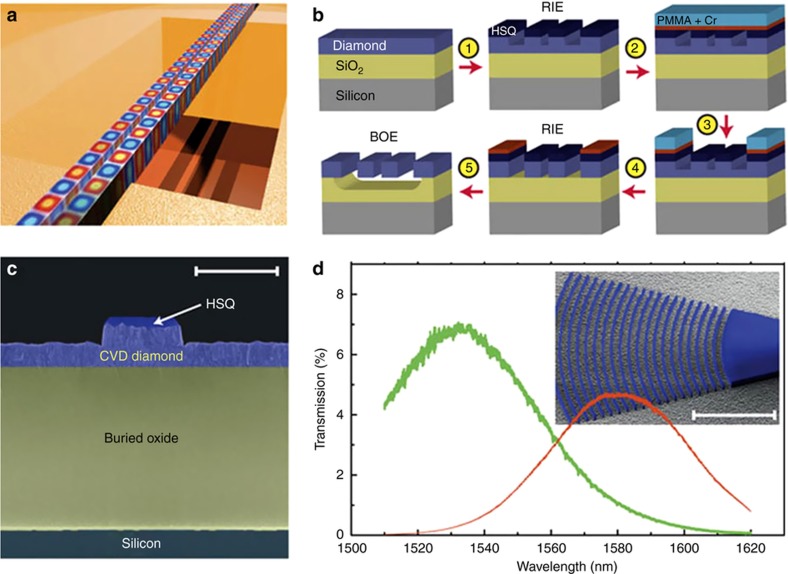
Diamond optomechanical resonators. (**a**) Schematic of coupled free-standing waveguides, which act as mechanical resonators. Propagating optical modes are overlaid in color. (**b**) Fabrication routine used to prepare both photonic circuitry and mechanical elements on-chip. (**c**) Cross-sectional scanning electron microscope (SEM) image of a diamond nano photonic ridge waveguide. Individual layers are marked in false-color. Scale bar, 1 mm. (**d**) Transmission curves of diamond waveguides connected to focusing grating couplers (inset: SEM image of a fabricated device, scale bar, 7.5 mm). The central coupling wavelength is tuned by adjusting the period of the grating. Images reprinted by permission from Macmillan Publishers Ltd: Nature Communications: Ref. 6. Copyright 2013.

Optomechanics is the study of the mechanical interaction between light and matter, mediated by radiation pressure. When the interaction is enhanced by introducing an optical resonator, it is called cavity optomechanics^11^. Due to the ability to detect tiny mechanical forces, opto-mechanical systems have applications for the detection of gravitational waves, and are useful experimental systems to test quantum mechanics and decoherence models^6^ (see Figure 11).

**Figure 12:**
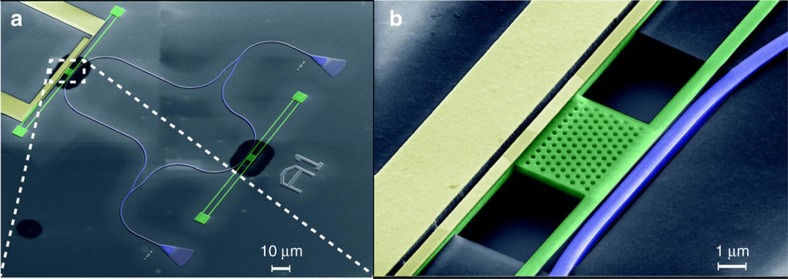
False color scanning electron microscope-micrographs of a fabricated electro-optomechanical device. (**a**) The integrated Mach-Zehnder interferometer is shown in blue. The mechanical resonator, which is evanescently coupled to the waveguide, is shown in green, while the metal electrodes are shown in golden color. (**b**) Detailed view of the free-standing resonator. The photonic crystal mirror separates the optical components from the electrode section. Images reprinted from Ref. 95, with the permission of AIP Publishing.

The detection of the actively modulated nano-mechanical response of up to 115 MHz at room temperature condition has been shown in integrated electro opto-mechanical circuits, featuring sensitive interferometric motion readouts and mechanical resonators with high *Q*_s_ up to 9600 using PCD^95^. The H-beam resonator is electrically driven on one side by metal electrodes, while the motion is detected on the opposite side by an optical Mach-Zehnder interferometer; the two systems are optically separated by a PHC engraved into the beam by EBL (see Figure 12). This enables opto-mechanical devices for a broad range of applications, such as optical actuators, vibration sensors and reconfigurable optical elements.

A single-crystal diamond opto-mechanical system was shown to excite mechanical oscillations in nano-beams with lengths from 50 to 80 μm, with amplitudes of more than 200 nm and a *Q*_m_ of 7·10^5^, allowing stress fields strong enough to potentially couple to the NV-center spin transitions over a 150-nm bandwidth with a coupling coefficient up to 45 GHz/nm and a displacement sensitivity of 9.5 fm·Hz^−1/2^ (Ref. 32).

The tunable coupling of single NV-center spins to external electromagnetic fields for quantum information applications was recently demonstrated in a hybrid spin-electro-mechanical device, where, by integrating a NV-embedded diamond beam with a superconducting coplanar waveguide cavity, a single NV-center spin is coupled to the single microwave cavity photons, enabling the mediation of coherent information transfer by mechanically dark polaritons^96^. In analogy with optical PHC, diamond crystals (OMCs) have been proposed, where a quasi-periodic diamond nanostructure leads to coupling of an optical cavity field to a mechanical mode via the radiation pressure of light. In contrast to other material systems, diamond OMCs operating in the resolved sideband regime possess large intra cavity photon capacity (>10^5^) and a sufficient opto-mechanical coupling rate to exceed a cooperativity of ~1 at room temperature^12,32,33^.

In the above examples, the NV spin ground state is coupled with mechanical modes and read out optically, whereas other methods are employed when the NV excited state is coupled to the cavity mechanical modes. NV-excited state presents a much stronger electron-phonon interaction compared to its ground state. This property makes it ideal for optomechanical control of the center quantum states via phonon-assisted optical transitions or sideband transitions, at the expenses of cryogenic operation. Further work in this direction at 8 K is contained in Ref. 97, where the NV excited-state electron-phonon interaction permit to couple its direct dipole optical transitions to surface acoustic waves, achieved on a patterned layer of piezoelectric ZnO, sputtered on a bulk diamond surface. This method permits the control of the internal electron states of the NV corresponding to the ground and excited state spin states and it can be extended to control the motional states of a coupled NV nano-mechanical system through these optomechanical processes.

## Conclusions and outlook

As discussed in this review, recent advances in diamond synthesis and nanofabrication have enabled high-quality nano-mechanical systems for force microscopy and nano-fluidics as well as nano-photonic devices for increased photon collection and tailored light-matter interaction. In the classical application of nano-cantilevers for AFM, diamond has proved to be better than Si, with a force sensitivity of 0.54 aN Hz^−1/2^ at 100 mK (Ref. 27). However, using thinner cantilevers to increase sensitivity, the mechanical-*Q* tends to be reduced due to surface friction.

A hybrid solution of a diamond nanowire with a Si cantilever provides the current record sensitivity of 2.4 aN·Hz^−1/2^ at 4 K (Ref. 37). However, this is not yet in the region of the zN range for nuclear magnetic resonance signals using spin magnetic resonance microscopy although projected values indicate the possibility.

The integration of NV center in diamond nano-cantilevers and nanopillars, and their combination, has advanced the field of diamond probes for scanning probe microscopy of magnetic fields, reaching resolutions of 25 nm for an AC magnetic field sensitivity of 50 nT·Hz^−1/2^ at room temperature. This is due to the improved coherence time of the NV spin in the nanostructures, based on high-purity single crystal diamond. The coupling of NV spin with the mechanical mode of a nano-cantilever permits the control of the NV spin coherence by induced strain and thus strain imaging via spin detection with a sensitivity of 3×10^−6^ strain·Hz^−1/2^. By reducing the mode volume of the cantilever’s mechanical mode, it is possible to increase the coupling strength between NV spin and diamond phonons to reach strong spin-phonon coupling and quantum coherence control of spins via mechanical dynamics. This opens new architectures for spin-spin interaction via phonons.

In optical and photonics applications, high Q_s_ up to 10^6^ have been obtained in ring resonators and up to 10^5^ in disk resonators at 1550 nm. However, due to large mode volumes, the maximum spontaneous emission enhancement tends to be low. In the visible region, *Q*-values tend to be much lower even for ring resonators, and, in this spectral region 1D-PHC cavities maximum *Q* is 24 000. The maximum coupling of single NV with a 1D-PHC is achieved with a *Q*=9900 with a loaded *Q*=3000 when strongly coupled to NV^51^, while it appears that the best performance in the direction to operate non-linear quantum optics and integrated optical quantum networks is achieved by entangling SiV color centers in diamond 1D nano-cavities^68^.

A combination of these advances has led to optomechanical devices in diamond, and we can foresee novel applications in spin optomechanics where optical and mechanical modes are coupled in the same devices with spin defects; additionally spin control can be achieved in diamond crystals by photonics approach at the expenses of operating at cryogenic temperature. While initial approach to cavity optomechanics was achieved in PCD, SCD provides lower mechanical dissipation and higher NV coherence, demonstrated in SCD microdisk^12^ with opto-mechanical cooperativity C~3 at room temperature. This is currently the direction of most expansion for diamond micro- and nano-mechanical devices applications for sensing, particularly with the promise of higher temperature operation. Another path to optomechanics could be the use of a bulk diamond at low temperature, where the Brillouin interactions are altered and a bulk crystal can be regarded as a coherent macroscopic opto-mechanical system, providing access to ultrahigh *Q*-factor (~10^7^/10^9^) and phonon modes at very high frequencies (12 GHz)^98^. However, in this case coupling with atomic color centers may result challenging and the low-temperature operation will limit sensing applications, while being an avenue for fundamental physics studies.

In summary, ideally, an integrated nanoscale mechanics or photonics platform in diamond should mimic the planar technologies which have been developed in SOI since the mid-1990s^99,100^, all operating at fiber optic telecommunication wavelength, *λ*~1550 nm. Despite the progress in diamond nanofabrication, there are still fundamental challenges to be overcome regarding their employment in some of the foreseen applications, particularly in the quantum technology domain where the nanofabricated structures need to have integrated color centers. The first limit is the lack of color centers in diamond for the infrared region.

Quantum photonic network implementations are based on diamond photonic nano-structures operating in the visible range, and the integrated color centers require long spin coherence times and lifetime limited emission line widths. In this case, more developments must be undertaken to produce nanostructures with both high intrinsic quality (high *Q* values and low mode volumes) and high defect quality to be able to operate at higher temperature. Similarly, application of integrated nanomechanical systems and nanosensors based on NV require a long coherence time. Some of the nanofabrication procedures, such as dry etching, can introduce degradation and increase the surface area near defects, leading to the degradation of defect properties. For this reason, proper surface termination methods must be developed. Furthermore, the realization of large-scale quantum photonic systems will depend on scalable fabrication techniques and on the tunability to match the resonance wavelength with the defect transition frequency without degrading the cavity *Q*. Finally, following the recent success with diamond, other materials hosting color centers with similar quantum properties are emerging, with suitable infrared operation, low-cost single crystal fabrication and CMOS compatibility such as SiC^101^, or 2D materials, which could provide a higher light confinement and control if integrated in nanostructures^102^, albeit these materials have not reached the same level of maturity in the here targeted applications.

## Author contributions

SC and AB designed the manuscript, processed the references, prepared the original figures and wrote the draft and manuscript. LR, JB and MZAA contributed to the discussion and the further refinement of the manuscript.

## Figures and Tables

**Table 1:** Properties and geometry of singly-clamped nanobeams from electronic-grade and optical-grade single crystal diamond, with rectangular and triangular cross sections

Length (μm)	Width (μm)	Thickness (nm)	*Q*_m_	*f*_c_ (MHz)	*f*×*Q*_m_ (THz)
4.8	0.82	530*	47, 000	40	1.88
9.6	1.3	1, 150*	21, 000	28.6	0.6006
9.6	0.82	530*	77, 000	10.4	0.8008
9.9	1.040	437*	55, 000	9.1	0.54
14.7	0.82	530*	92, 000	4.7	0.4324
19	0.82	530*	100, 000	2.7	0.27
24	0.82	530*	100, 000	1.7	0.17
29	0.82	530*	100, 000	1.2	0.12
34	0.82	530*	120, 000	0.89	0.1068
38	0.82	530*	145, 000	0.686	0.09947
43	0.82	530*	150, 000	0.539	0.08085
48	0.82	530*	140, 000	0.411	0.05754
60	15	1, 700	54, 000	1.39	0.07506
60	15	1, 400	129, 000	1.146	0.147834
60	15	1, 100	128, 000	0.89	0.11392
60	15	760	222, 000	0.637	0.141414
80	20	2, 100	133, 000	1	0.133
80	20	1, 800	165, 000	0.843	0.139095
80	20	1, 500	338, 000	0.728	0.246064
80	20	1, 200	100, 000	0.586	0.0586
240	12	280	380, 000	0.13	0.0494
240	12	660	412, 000	0.32	0.13184

* indicates triangular cantilevers.
